# Deviated Nasal Septum a Risk Factor for the Occurrence of Chronic Rhinosinusitis

**DOI:** 10.7759/cureus.30261

**Published:** 2022-10-13

**Authors:** Shivani N Malpani, Prasad Deshmukh

**Affiliations:** 1 Otolaryngology, Datta Meghe Institute of Medical Sciences (Deemed to be University), Wardha, IND

**Keywords:** computed tomography, ostiomeatal complex, paranasal sinus diseases, chronic rhinosnusitis, deviated nasal septum

## Abstract

The aim of this review article is to determine whether a deviated nasal septum (DNS) is a potential risk factor for the occurrence of chronic rhinosinusitis (CRS). Nasal septal deformities include spur, deviated nasal septum, thickening, and dislocation. Deformities of the nose tip and columella are examples of external deformities, together with the deviated part of either cartilage or both the bony and cartilage part of the dorsum of the nose. Various symptoms of chronic rhinosinusitis include nasal obstruction, nasal or post-nasal drainage, facial pain and pressure, and smell disturbances. For a long time, the deviation of the nasal septum is related to the pathogenesis, progression, and severity of chronic rhinosinusitis. Mechanisms involving mechanical and aerodynamics theory may be used to explain this relationship. Etiology in the occurrence of CRS are allergy, asthma, tooth Infection, immunodeficiency, mucociliary disorders, anatomical irregularities like DNS, concha bullosa, septum spurring, or an expanded cystic middle turbinate or prominent agger nasi cells that compromise the osteomeatal complex. The computed tomography (CT) scan imaging of the nasal cavity and paranasal sinuses has dramatically improved especially since the use of coronary CT scans. These scans make it simple to find even minute changes and abnormalities in bony structures and mucosal pathologies. An increase in the angle of DNS is significantly linked to specific disease patterns in the osteomeatal complex. This review shows that not all subtypes of DNS always result in the development of CRS. Only extremely severe DNS appears to contribute to the etiology of CRS.

## Introduction and background

**Figure 1:**
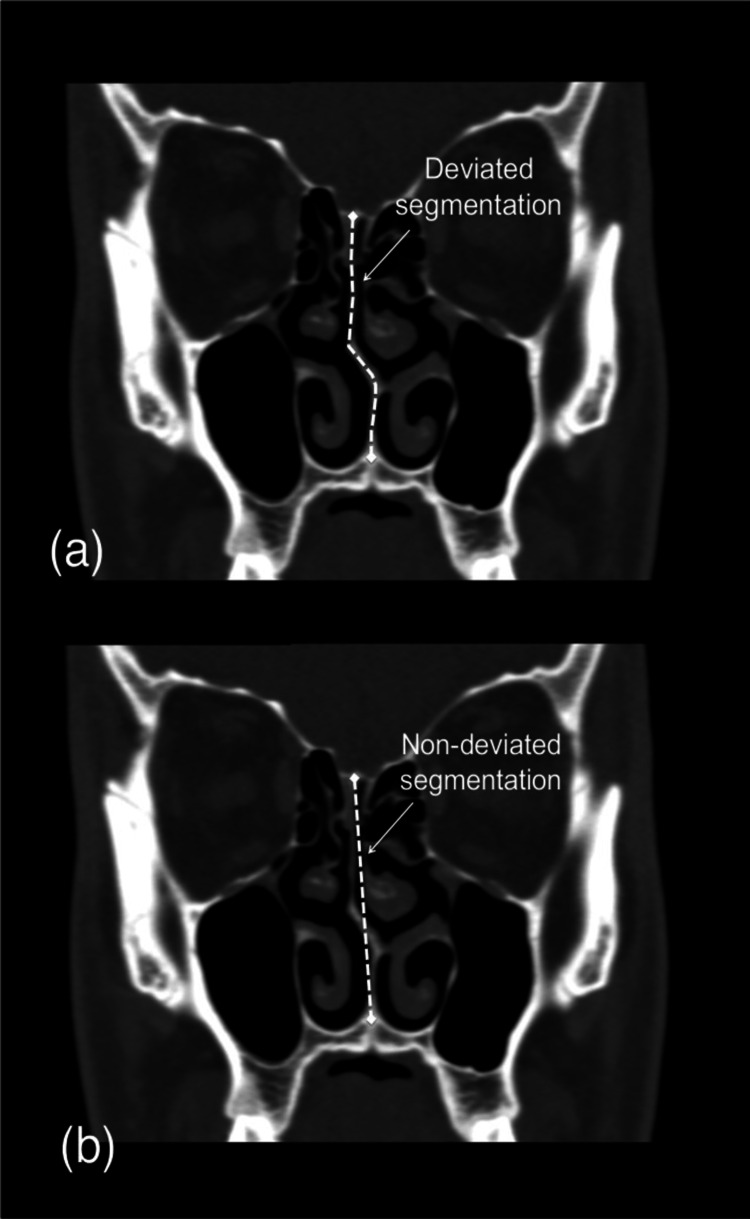
The CT scan of the nasal septum (a) Deviation of the nasal septum, (b) Normal nasal septum Image sourced from open access journal under a CC-BY license, contributed by Hartman et al. [6].

The septum proper comprises the columellar septum (formed by the medial crura of the alar cartilage) and the membranous septum (consists of a double layer of skin). There is no bony or cartilaginous support to the nose. The septum proper, which consists of an osteocartilaginous framework, is made up of its principal constituents which are the perpendicular plate of the ethmoid, vomer, the septal quadrilateral cartilage, and other bones such as the crest of the nasal bone, palatine bone, and maxilla. The nasal cavity’s cartilage and erectile tissue, mainly consisting of septal cartilage and inferior conchal cartilage, serve as valves in the nasal cavity and regulates airflow in the nasal cavity. The narrowest part of the nasal cavity in the anterior area which is from the nasal valve to the nostrils has the highest nasal airflow resistance. This is crucial for the functioning of the nose and also for the principal symptom which is nasal congestion [1-3]. The septal deformities include spurring, deviated nasal septum, thickening, and dislocation. The deviation of the nasal septum often raises complaints such as nasal congestion, headache, anosmia, sinusitis (because of obstruction of sinus ostia), and epistasis. Various symptoms of chronic rhinosinusitis (CRS) include nasal obstruction, nasal or post-nasal drainage, facial pain, and pressure and smell disturbances. One in seven individuals with rhinosinusitis will result in an annual diagnosis of a minimum of 50 million new cases having rhinosinusitis worldwide. Chronic rhinosinusitis is the fifth most frequent diagnosis for which antibiotics are prescribed in adults, receiving more than one in five antibiotic prescriptions [4]. The normal anatomy of cartilage, bone, or both may be affected by a deviated nasal septum (DNS) (Figure 1). Different types of DNS exist, including anterior dislocation, C-shaped deformity, S-shaped deformity, spur formation, and thickening (which might be due to localized hematoma or overlapping of displaced fragments). Males are more frequently affected than females by DNS, however, it can affect people of any age and sex [5]. 

## Review

Classification of the deviated nasal septum

As per Cottle's classification, the degree of septal deviation could indicate simple DNS, obstructed DNS, or impacted DNS. As per the site, DNS could be classified as anterior or cartilaginous deviation/posterior or bony deviation. Depending on the involvement of the external nose, DNS could be deemed with or without external deviation. And finally, based on progression, DNS could be determined to be asymptomatic or symptomatic.

Septal mucosa changes in the DNS

The septum mucosa is formed by the basement membrane, lamina propria, and the epithelium lining (ciliated columnar cells). In DNS, due to the changes in the airflow, dynamics histopathological variations in the nasal mucosa like squamous metaplasia and lymphocyte infiltrating in mucosa may be seen. The severity of these variations is more pronounced on the concave side of the nasal cavity. It has been reported that there is severe loss of cilia and prolonged saccharin clearance time on the concave side in comparison with the convex side. Thus, in the histopathological study, the concave side shows a more intensive infiltration of inflammatory cells and a less dense distribution of serous and mucinous glands [7]. According to additional theories, there is a significant degree of changes in the mucosa of the concave side in comparison to the convex side [8,9]. According to other authors' studies, there is mucosal thinning on the convex side of the DNS [10,11]. This is due to the mechanical forces that misalign the cartilaginous part or causes the spurring of the nasal bone. Thus, it is especially crucial to take utmost precaution while lifting the mucoperichondrium part of the septum's convex side during surgical repair of the DNS. Mucus contributes to maintaining physiological homeostasis because of its exceptional chemical and physical properties. When this environment is disturbed, rhinitis, rhinosinusitis, and some other upper respiratory tract and lower respiratory tract disorder may appear [10,11].

Chronic rhinosinusitis

Definition

The term "chronic rhinosinusitis" is defined as nasal and paranasal sinus involvement causing inflammation of these structures [12].

Etiology

Either the anatomical, physiological or pathological alterationsprevent free drainage from the paranasal sinuses and cause stagnation of secretion thus, this increases the risk of infection. It has been suggested that various conditions contribute to the emergence of chronic rhinosinusitis. One of which is exposure to allergens which leads to mucosal edema of the nose, thus obstructing the sinus ostia and causing the formation of negative pressure. It also changes the pH to be less than seven, resulting in an alteration in mucociliary clearance. The above mechanism leads to impaired drainage and stasis of mucus in the sinuses. This predisposes to bacterial infection and leads to CRS.

**Figure 2:**
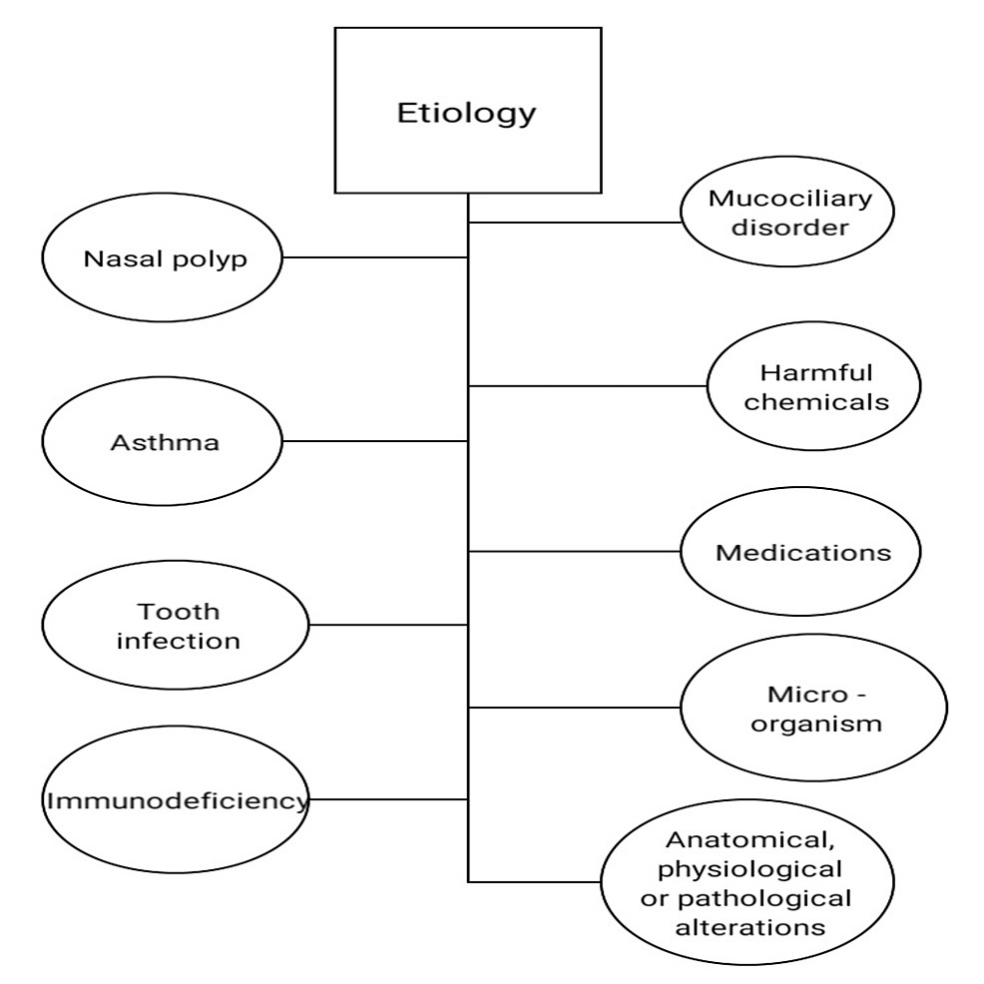
Etiology of CRS CRS: Chronic rhinosinusitis Image credit: Author Shivani N Malpani

The following plays a role in the genesis of CRS (Figure 2): asthma; tooth infection of premolars and molars (could cause maxillary sinusitis); nasal polyps; human immunodeficiency virus (HIV)-related illnesses (where opportunistic micro-organisms such as fungi, microsporidia, and pseudomonas aeruginosa could lead to CRS); mucociliary disorders such as cystic fibrosis, primary ciliary dyskinesia, and Kartegener’s syndrome that impair the clearance of sinus secretion and lead to inflammation of the paranasal sinuses; medications such as steroids, chemotherapy, immune suppressant, and nasal sprays; harmful chemicals from environmental pollution or occupational chemicals, dust and noxious gases; microorganisms (viral, bacterial, and fungal) like *Staphylococcus aureus *that produces toxins that cause inflammation in paranasal sinus mucosa.

Osteomeatal complex 

**Figure 3:**
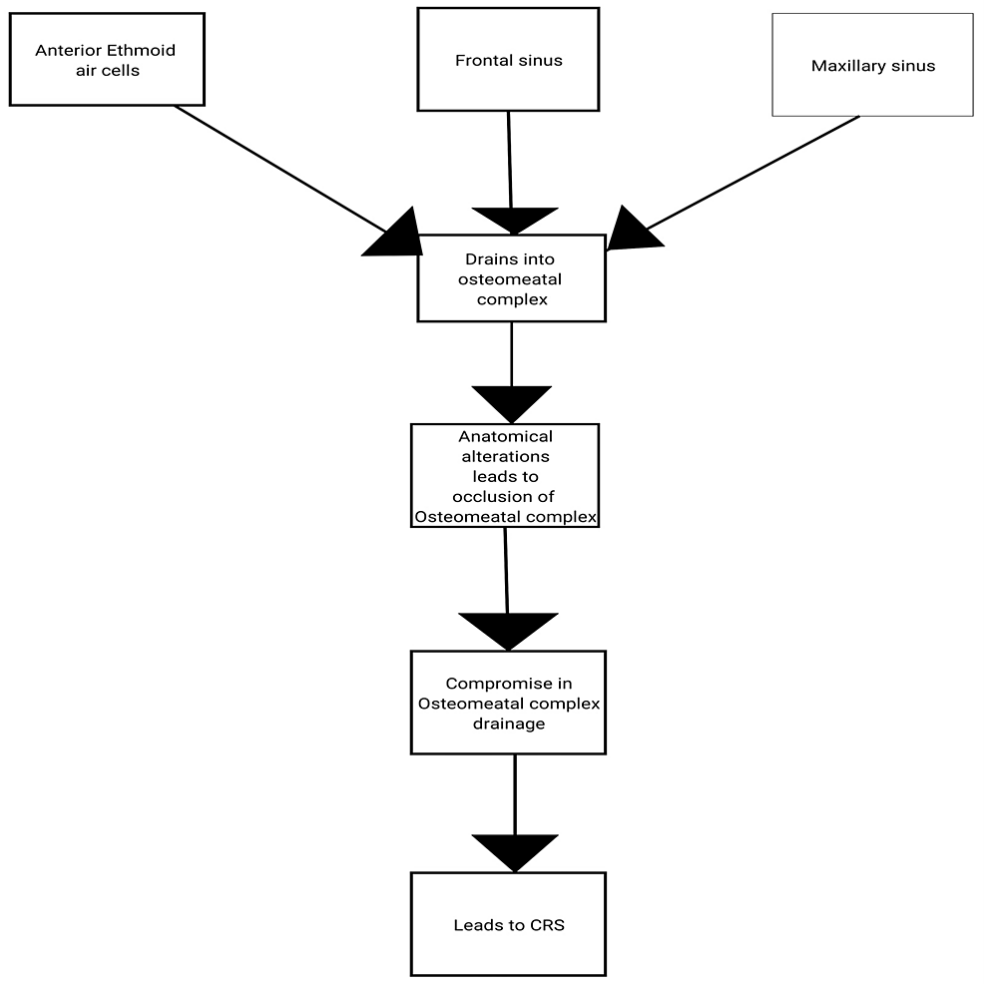
Osteomeatal complex drainage CRS: Chronic rhinosinusitis Image credit: Author Shivani N Malpani

The frontal, maxillary and anterior ethmoid air cells ultimately share a common channel for drainage and ventilation, which is represented by the osteomeatal complex. The osteomeatal complex is the space of the middle meatus which is below and lateral to the middle turbinate. One of the leading causes of CRS is the occlusion of this small area. Ventilation, drainage, and the lining mucosa of the nasal cavity are the three most crucial elements that determine the health and healthy functioning of the paranasal sinuses. A patent sinus ostium (pre-chamber) connecting the ostium to the nasal cavity is necessary for proper ventilation of the paranasal sinuses [13-15]. Anatomical alterations like a DNS, concha bullosa, septum spurring, or an expanded cystic middle turbinate or prominent agger nasi cells compromise the osteomeatal complex (Figure 3).

Factors for CRS

Non-allergic rhinitis with eosinophilic syndrome (NARES)-nonallergic rhinitis with eosinophilia syndrome includes symptoms like dysosmia, nasal obstruction, and nasal discharge. Staphylococcus and its enterotoxin cause stimulation of the T helper type 2 (Th2) system that promotes immunoglobulin E (IgE) production. This also contributes to the eosinophilic infiltration in the septal mucosa.

Vasomotor rhinitis, rhinogenic disorders, and odontogenic infections lead to type 2 inflammation and this is considered to be the hallmark in the pathogenesis of allergy. In sensitized patients, aeroallergen is recognized by nasal dendritic cells. This activates the helper T lymphocytes and then these cells migrate to the bone marrow. This process results in the release of cytokines interleukin (IL)-4, IL-5, and IL-13, thereby stimulating the formation of eosinophils, mast cells, and basophils. These cells enter the systemic circulation and recognize adhesion molecules and chemotactic signals.

Structural abnormality

A block in the osteomeatal complex or the opening of sinuses results in impaired ventilation of sinuses, impaired mucociliary clearance, immune dysfunction, impaired epithelial defense, and biofilm formation. The most commonly affected sinus is the maxillary followed by ethmoidal, sphenoidal, and frontal.

Diagnosis

In patients with mucosal edema and nasal discharge, a nasal endoscopic examination could reveal nasal polyps and mucopurulent discharge. Microbiological swabs taken from this mucopurulent discharge are used to detect the causative micro-organism. Also, endoscopy detects the presence of all anatomical variations like a large turbinate or a DNS. The CRS finding on a sinus CT scan is determined by the sinus opacity caused by enlargement of the sinus mucosa and/or residual secretion. Paranasal sinus CT scansare performed in the axial, coronal, and sagittal planes. An MRI scan with contrast can also be used.

For the clinical diagnosis of CRS, laboratory testing such as nasal cytology, nasal biopsy, and hematologic analysis is not required. However, they can help identify the presence of additional illnesses, such as an allergy or an acute bacterial infection as well as more serious conditions such as cystic fibrosis, ciliary dysfunction, or immunodeficiency [12].

Treatment of CRS

Antibiotics

Chronic sinusitis is frequently treated with cephalosporin antibiotics. The course of this treatment is more than 12 weeks for CRS. Third-generation cephalosporins are prescribed one or two times a day. Cefdinir and cefpodoxime are the third generation of choice [16]. There are currently specific indications for the use of fluoroquinolones in the treatment of sinusitis in adults [17]. Amoxicillin potassium clavulanate is a suitable option as an antibiotic for acute episodes of chronic rhinosinusitis. According to some specialists, patients should receive care for a total of seven days after their symptoms have disappeared [18]. 

Decongestants

Decongestants are α-adrenergic agonists that have a negligible impact on the improvement of symptoms like nasal blockage or nasal congestion and the reduction of nasal airway resistance. Only phenylephrine and pseudoephedrine are frequently used as oral decongestants. Both α1 or α2 adrenergic receptor activation by direct-acting sympathomimetics causes vasoconstriction and reduction in nasal congestion.

Glucocorticosteroids

The most effective and commonly employed medical treatment is corticosteroid sprays. After the nasal antigen challenge, topical corticosteroids have a definite impact in causing the inflow of inflammatory cells into the nasal mucosa thereby stimulating an immune response [19]. It has been demonstrated that pretreatment of the nasal mucosa with an inhaled steroid alters both the early and late phases of the immune response to the antigen nasal challenge test [20]. Glucocorticoids also prevent antigen-induced histamine hyperresponsiveness. Inhaled corticosteroids are effective adjuvant treatment options for chronic rhinosinusitis. This is because they are safe anti-inflammatory agents and also help in relieving nasal congestion. Commonly used corticosteroid nasal drops are betamethasone and fluticasone.

Adjunctive Therapies

Saline helps keep nasal secretions from crusting, especially around the osteomeatal complex. Steam moistens the dry, irritated mucosa while liquefying and softening crusts. Mucolytics break down mucus, liquefy it and facilitate the removal of mucus. Astringents such as pine oil, eucalyptus oil, and mentholated products like Vicks VapoRub enhance the therapeutic effects of the steam treatment.

Expectorant

Expectorants cause gastric irritation and cause reflex irritation of the bronchi causing gradual removal of secretion from the respiratory tract. Guaifenesin is used as an expectorant. When the respiratory tract is congested and filled with mucus, guaifenesin is used in symptomatic management.

*Intravenous Immunoglobulin (*IVIG)

Immunodeficiency is a risk factor in causing CRS. For antibody deficiency illnesses and common variable immunodeficiency, IVIG is approved as a replacement therapy [21].

Surgical Approaches

**Figure 4:**
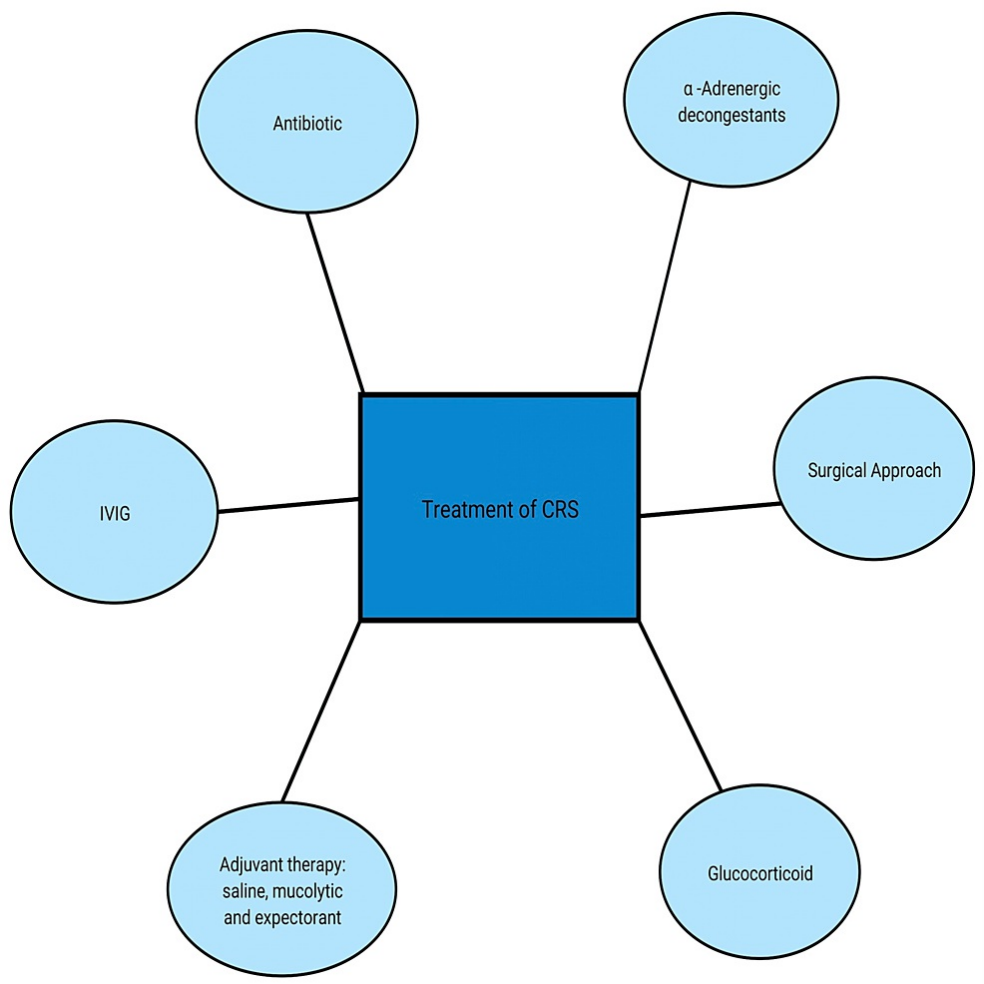
Treatments available for CRS CRS: Chronic rhinosinusitis, IVIG: Intravenous immunoglobulin Image credit: Author Shivani N Malpani

The surgical gold standard for treating chronic infectious rhinosinusitis now typically involves endoscopic techniques such as functional endoscopic sinus surgery (FESS). A complication of FESS is hemorrhage or injury to any of the structures such as the cribriform plate, lamina papyracea, onodi cells, sphenoid sinus, and ethmoidal artery. Considering the size and sinus involvement, open surgical operations are still needed i.e., frontal or sphenoid. Patients with acute or chronic frontal rhinosinusitis should be monitored while undergoing trephine and postoperative irrigation (Figure 4).

Role of the CT scan in diagnosing CRS

Nasal and paranasal sinuses are properly visualized on a CT scan. The development of coronary CT scans used in imaging of the paranasal sinus led to a dramatic improvement in the diagnosis of CRS [22]. This method makes it simple to find even minute changes and abnormalities in bony structures and mucosal pathologies. For a long time, it was believed that the pathogenesis, progression, and severity of developing CRS were related to the deviation of the nasal septum. The definition of DNS is the curving of the nasal septum morphology on a coronal CT scan. The nasal septal curvature's convexity served as a description of the direction of deviation [23,24]. Even a comparatively new study has verified this connection, so it is advised that people with persistent symptoms and recurring rhinosinusitis undergo a close examination of their CT scan findings [25]. On the CT scan, the degree of DNS and other abnormalities linked with rhinosinusitis can be assessed. The presence of mucosal alterations, from mild mucosal thickening to complete sinus opacification, as well as significant clinical symptoms were taken as indicators of abnormality. According to certain research, an increase in the angle of deviation in the nasal septum in the osteomeatal complex area is directly linked to severity and also the occurrence of paranasal sinusitis. But in these cases, anatomical variations connected to osteomeatal complex were complicit [26-30].

The mechanism through which DNS leads to the occurrence of CRS

**Figure 5:**
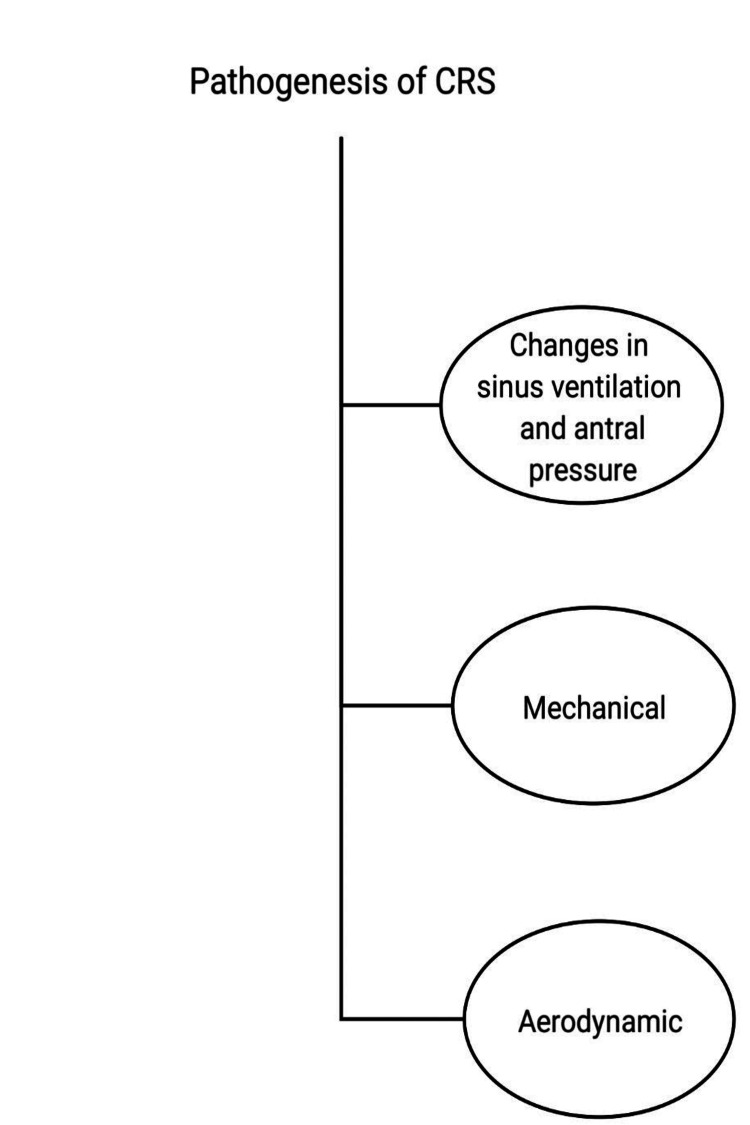
Pathogenesis of CRS CRS: Chronic rhinosinusitis Image credit: Author Shivani N Malpani

This review (Table 1) offers three physiopathological ideas (Figure 5) explaining how DNS leads to rhinosinusitis: 1) changes in sinus ventilation and antral pressures; 2) mechanical; 3) aerodynamic. According to the first theory, deviation of the posterior nasal septum leads to variations in antral pressure and airflow changes, leading to changes in paranasal sinus ventilation. The mechanical theory, which is the second theory, contends that secretions build up in the paranasal sinus as a result of the osteomeatal complex becoming narrower. Stagnated mucus secretion gets infected, leading to chronic rhinosinusitis. The third explanation, known as the aerodynamic theory, attributes the genesis of chronic rhinosinusitis as a result of alteration in the flow of air pressure in the paranasal sinus as well as the disruption in mucociliary clearance [31].

**Table 1:** Various theories indicating DNS as a risk factor for CRS CRS: Chronic rhinosinusitis, DNS: Deviated nasal septum

Studies on the link between DNS and CRS	
Researchers	Theory
Kennedy et al., 1985 [32]	Investigated the sinus mucociliary clearance. Whenever two mucosal surfaces came into contact there was a localized interruption of mucociliary clearance, which caused secretions to be contained in the zone of communication between surfaces thus increasing the risk of infection.
Collet et al.,2001 [33]	The nasal septum neither played a clear role in the development of chronic rhinosinusitis nor was it a contributing factor.
Gencer et al., 2013 [23]	Maxillary sinus volumes may be greater on the side opposite to septal abnormalities, according to Gencer et al. Additionally, there was a noticeably higher likelihood of discovering maxillary rhinosinusitis on the side opposite the substantial septum deviation.
Moorthy et al., 2014 [34]	Even in the absence of any nasal symptoms, "S" shaped DNS had a statistically significant link with paranasal sinus disease.
Rehman et al., 2012 [35]	When nasal septal obstruction and deformity were present, the majority of patients exhibited paranasal sinus disease. For the vast majority of individuals with nasal septal obstruction and deformity, Rehman et al. documented paranasal sinus pathology.
Mohebbi et al., 2012 [36]	In an epidemiological study of factors associated with DNS by CT scan, which was conducted in a 2012 study and which is a cross-sectional study, Mohebbi et al. found no association between the degree of septal deviation and the severity of sinusitis or osteomeatal involvement.

## Conclusions

There are two complementary techniques for inspecting the nasal cavity and paranasal sinus: nasal endoscopy, and CT scan of the paranasal sinus coronal view. According to aerodynamics, disturbances in antral ventilation and a mechanical obstruction may contribute to ipsilateral CRS. Contralateral CRS, however, could result from two other mechanisms apart from mechanical ones. These confirm the mechanical and aerodynamic impacts of severe DNS through the link between CRS and severe DNS. Also, not all subtypes of DNS always result in the development of CRS. Only extremely severe DNS appears to contribute to the etiology of CRS. Mechanisms involving mechanics and aerodynamics are used to explain this relationship. 
